# Efficient expansion and delayed senescence of hUC-MSCs by microcarrier–bioreactor system

**DOI:** 10.1186/s13287-023-03514-1

**Published:** 2023-10-04

**Authors:** Xia Wang, Liming Ouyang, Wenxia Chen, Yulin Cao, Lixin Zhang

**Affiliations:** 1 State Key Laboratory of Bioreactor Engineering, East China University of Science and Technology, Shanghai, 200237 People’s Republic of China; 2 Beijing Tang Yi Hui Kang Biomedical Technology Co., LTD, Beijing, 100032 People’s Republic of China

**Keywords:** Human umbilical cord mesenchymal stem cells, Microcarrier, Transcriptome, Cell senescence, Bioreactor, Cell culture

## Abstract

**Background:**

Human umbilical cord mesenchymal stem cells (hUC-MSCs) are widely used in cell therapy due to their robust immunomodulatory and tissue regenerative capabilities. Currently, the predominant method for obtaining hUC-MSCs for clinical use is through planar culture expansion, which presents several limitations. Specifically, continuous cell passaging can lead to cellular aging, susceptibility to contamination, and an absence of process monitoring and control, among other limitations. To overcome these challenges, the technology of microcarrier–bioreactor culture was developed with the aim of ensuring the therapeutic efficacy of cells while enabling large-scale expansion to meet clinical requirements. However, there is still a knowledge gap regarding the comparison of biological differences in cells obtained through different culture methods.

**Methods:**

We developed a culture process for hUC-MSCs using self-made microcarrier and stirred bioreactor. This study systematically compares the biological properties of hUC-MSCs amplified through planar culture and microcarrier–bioreactor systems. Additionally, RNA-seq was employed to compare the differences in gene expression profiles between the two cultures, facilitating the identification of pathways and genes associated with cell aging.

**Results:**

The findings revealed that hUC-MSCs expanded on microcarriers exhibited a lower degree of cellular aging compared to those expanded through planar culture. Additionally, these microcarrier-expanded hUC-MSCs showed an enhanced proliferation capacity and a reduced number of cells in the cell cycle retardation period. Moreover, bioreactor-cultured cells differ significantly from planar cultures in the expression of genes associated with the cytoskeleton and extracellular matrix.

**Conclusions:**

The results of this study demonstrate that our microcarrier–bioreactor culture method enhances the proliferation efficiency of hUC-MSCs. Moreover, this culture method exhibits the potential to delay the process of cell aging while preserving the essential stem cell properties of hUC-MSCs.

**Supplementary Information:**

The online version contains supplementary material available at 10.1186/s13287-023-03514-1.

## Background

Mesenchymal stem cells (MSCs) have been widely used in stem cell therapy due to their self-renewing immunomodulatory properties, maintenance of tissue homeostasis, and pluripotent differentiation ability, and have attracted more and more attention in the field of regenerative medicine [1, 2]. Indications of MSCs therapy to date include acute lung injury, diabetes, myocardial infarction, graft-versus-host disease (GVHD), aplastic anemia, arthropathy, liver disease, spinal cord injury, systemic lupus erythematosus, and stroke [3, 4].

With the range of indications continue to expand, large-scale in vitro amplification of MSCs becomes a prerequisite for such therapeutic interventions. The most used method for MSCs expansion at present is artificial planar subculture. However, it is labor-intensive, susceptible to contamination, and lacks in-line process monitoring and control, resulting in poor batch-to-batch quality stability [5].

Bioreactor systems provide a solution for engineered expansion of MSCs. Different bioreactor systems have been tried in the culture of MSCs, including fixed-bed bioreactors [6, 7], stirred suspension bioreactors, perfusion bioreactors [8, 9], or hollow fibers [10]. Among them, microcarrier-based stirred bioreactor cell culture facilitates high-density culturing of MSCs in suspension, due to its high surface-to-volume ratio [11], flexible handling, controlled culture conditions [12], comprehensive process parameters including dissolved oxygen, viable cell number, pH, temperature, and metabolites, etc. Therefore, a growing number of studies are using microcarrier-based suspension culture to amplify human MSCs (hMSCs) [13, 14].

During planar culture, the aging-related phenotype of cells increases as the number of passages increases, and the therapeutic efficacy gradually decreases [15]. hMSCs cultured in microcarrier–bioreactors are exposed to a significantly different microenvironment than in planar cultures, including shear stress caused by impeller stirring, changes in adhesion surfaces, etc. [16]. How about the senescence state and biological characteristics of MSCs cultured under microcarrier–bioreactor conditions? Several studies have reported an increase in the expansion rate of MSCs in bioreactor systems, along with changes in surface marker expression, colony formation capacity, and differentiation capacity [12, 17, 18]. However, there is a lack of comprehensive research focusing on the global gene expression profiles. By conducting transcriptome comparative analysis, we can gain a better understanding of the molecular mechanisms underlying the differences in cell phenotypes caused by the two culture methods. In this study, hUC-MSCs were simultaneously expanded using continuous planar culture and a bioreactor with self-made microcarrier. The cellular phenotypes were compared, and RNA sequencing (RNA-seq) was performed to generate global gene expression profiles. The comparison of cell phenotypes demonstrated differences in proliferative capacity, cell cycle states, and degree of cellular aging between hUC-MSCs amplified through the two culture methods. Transcriptome analysis revealed molecular mechanisms associated with cellular senescence and identified significant differences in the cellular gene expression profiles in response to the two distinct culture methods. These results provide support for the efficiency and quality of our self-made microcarrier–bioreactor process in the amplification of hUC-MSCs.

## Methods details

### Cell

Human umbilical cord mesenchymal stem cells are provided by Beijing Tang Yi Hui Kang Biomedical Co., Ltd. The procedure for collecting tissues was approved by the Medical Ethics Committee of Jilin Guojian High-tech Maternity Hospital.

### Planar culture

Cells were incubated at 37℃, 5% CO_2_ with serum-free media (Applied Cell, Shanghai, China) changes every 3 days. Cells were passaged once the monolayer reached 85% confluence and continuously cultured to 12 passages. Four generations of cells (P3, P5, P9, P12) were sampled from a continuous culture for transcriptome sequencing with three replicates per sample. The morphological changes of hUC-MSCs in each generation were monitored by examining them under an inverted microscope (Nikon).

### Microcarrier–bioreactor culture and harvesting

After the fusion degree of P6 generation cells reached 85% in plane culture, the cells were harvested. The harvesting process involved aspirating the medium, followed by a single wash with 10 mL Dulbecco’s phosphate-buffered saline (DPBS). Subsequently, the cells were lifted from the substrate with TrypLE (Invitrogen, USA). The cell number, cell viability, and cell size were determined with the Countstar Rigel S2 fully automatic fluorescence cell analyzer (Rui yu-biotech, Shanghai, China) using AOPI staining kit. The harvested P6 cells were continuously cultured by the bioreactor for three batches, named BioR1 to BioR3. The batch culture method is as follows: hUC-MSCs were cultured in 500-mL bioreactor (Tang Yi Hui Kang, Shanghai, China) with 200 mL serum-free medium and 20 mL microcarriers [19] (Tang Yi Hui Kang, Beijing, China). The cells were seeded at 1 × 10^5^ cells/mL microcarriers. During the first 24 h, intermittent agitation was employed at a rotational speed of 35 rpm, with a stirring duration of 45 min followed by a 15-min pause, to facilitate cell adhesion on the microcarrier. Once the initial 24-h period elapsed, continuous mixing was implemented to prevent microcarrier agglomeration. After 6 days of culture, the cell convergence on a single microcarrier reaches 85% and more than 85% of the microcarriers in the bioreactor achieve this level of convergence. Then all the cells were harvested by following the subsequent steps: The stirrer speed was set to 0 rpm to allow the microcarriers to settle, and the culture medium was carefully removed. The microcarriers were then washed twice with prewarmed DPBS and incubated with the same amount of prewarmed TrypLE at 37 °C and 5% CO_2_ for 10–15 min. During the incubation, the microcarriers were gently shaken every 5 min to facilitate cell detachment. The TrypLE reaction was halted by adding fresh and prewarmed culture medium. Subsequently, the cells were separated from the microcarriers by using a cell strainer with a pore size of 75 μm and rinsed with DPBS. After centrifugation at 200 ×*g* for 5 min the supernatant was discarded, and the cells were resuspended in fresh and prewarmed medium to determine cell number, viability, and size. The resuspended cells of the appropriate concentration were then inoculated onto fresh microcarriers for culture of next batch in bioreactor. Three continuous batches of cells (BioR1, BioR2, BioR3) were sampled for transcriptome sequencing with three replicates per sample.

### Determination of cell population-doubling level (PDL)

Planar cultures underwent passaging every three days in accordance with the culture duration, and their PDL was determined by considering the seedling cell count and the harvested cell count. In comparison, microcarrier–bioreactor cultures undergo harvesting every six days, and their PDL is calculated using the same procedure. PDL is calculated as follows [20]:

$${\text{PDL}} = 3.32\left( {\lg X_{{\text{f}}} \, - \lg X_{{\text{i}}} } \right) + S$$

where *X*_*f*_ is the final viable cell count, *X*_i_ is the initial seeded cell count, and *S* is the PDL at the beginning of culture.

### Assessment of stem cell differentiation ability

The mesodermal differentiation potentials of hUC-MSCs during senescence were evaluated through a 3-week incubation of hUC-MSCs with adipogenic, osteogenic, or chondrogenic differentiation medium (Applied Cell, Shanghai, China). Subsequently, adipogenic-, osteogenic-, and chondrogenic-differentiated hUC-MSCs were subjected to two washes with DPBS and fixed with 4% paraformaldehyde (PFA) (Macklin, Shanghai, China) for 15 min at room temperature. The fixed and differentiated cells were then washed with PBS and stained at room temperature for 1 h with 2% Oil Red O, 2% Alizarin Red S, or 1% Alcian Blue solution (Applied Cell, Shanghai, China) to assess the levels of adipogenicity, osteogenicity, and chondrogenicity, respectively.

### Detection of cell levels of senescence-associated β galactosidase (SA-β-gal) and reactive oxygen species (ROS)

The cells were subjected to the specified treatments, and the SA-β-gal activity was assessed using the SA-β-gal Staining Kit (Beyotime, Nanjing, China), following the manufacturer's instructions. The levels of ROS were determined using the ROS assay kit (Solarbio, Beijing, China), following the manufacturer's instructions. Red fluorescence emitted as a result of dihydroethidium bromide oxidation was employed to quantify the level of superoxide radical (O2•-) level in the cells. For each sample, 1 × 10^4^ cells were harvested from the gated cells and fluorescence was immediately measured using flow cytometer (BriCyte E6, Shenzhen, China).

### Detection of cell cycle

Cell cycle analysis was performed using the cell cycle kit (Beyotime, Shanghai, China) according to the manufacturer's instructions. In simple terms, the cells were cultured as described above, collected, cleaned, re-suspended in cold PBS, fixed at 4 °C with 70% ethanol for more than 24 h. The cells were then centrifuged, re-suspended in cold PBS and stained with PI at 37 °C for 30 min. Then, the cells were detected by flow cytometry (BriCyte E6, Shenzhen, China) and the results were analyzed by FlowJo software (BD Biosciences).

### Identification of cell surface markers

hUC-MSCs were phenotypically characterized by flow cytometry. The hUC-MSCs (5.0 × 10^4^ cells) from the 6 experimental groups were incubated with fluorescein isothiocyanate (FITC)-, phycoerythrin (PE)-, or allophycocyanin (APC)-conjugated monoclonal antibodies against isotype-APC, isotype-PE, isotype-FITC, CD19, CD90, CD105, CD73, and HLA-DR (BioGems, CA) for 30 min at 4 °C. The cell populations were analyzed using a FACScan instrument (BriCyte E6). As a control, non-treatment hUC-MSCs and isotype-PE and isotype-FITC or isotype-APC Ig control for each wavelength were used. Data were analyzed using FlowJo software (BD Biosciences).

### RNA extraction and library construction

The samples were extracted for total RNA using TRIzol reagent, and the measurement of RNA integrity and total volume was taken accurately using the Agilent 2100 Bioanalyzer system. RNA libraries were amplified for each sample following the Illumina protocol. Once the library inspection confirmed its quality, the libraries were pooled based on specified criteria for effective concentration and target data volume. Subsequently, the samples were subjected to sequencing using the Illumina NovaSeq 6000, generating 150-bp paired-end reads (performed by Novogene, Beijing, China).

### Analysis of differentially expressed genes and their functional enrichment analysis

Differential expression analysis of two groups (three biological replicates per condition) was performed using the DESeq2 R package (1.20.0). DESeq2 provides statistical routines for determining differential expression in digital gene expression data using a model based on the negative binomial distribution. The resulting P-values were adjusted using the Benjamini and Hochberg’s approach for controlling the false discovery rate. *p*-adjusted value (*padj*) <  = 0.05 and |log2(foldchange)|> = 1 were set as the threshold for significantly differential expression. Gene ontology (GO) and KEGG pathway enrichment analysis of the differentially expressed genes (DEGs) were performed by clusterProfiler (version 3.8.1) software. The enrichment threshold for GO term and KEGG pathway analysis was defined as a *padj* lower than 0.05 for the DEGs.

### GSEA

Gene Set Enrichment Analysis (GSEA) is a computational method used to assess whether a pre-defined set of genes exhibits a consistent and significant difference between two biological states. This approach involves ranking genes based on their degree of differential expression in two samples. Subsequently, the predefined gene sets were evaluated to determine whether they are enriched at either the top or bottom of the ranked gene list. GSEA allows for the detection of subtle changes in gene expression. In our analysis, we utilized the local version of the GSEA tool from the Broad Institute (http://www.broadinstitute.org/gsea/index.jsp). GO and KEGG data sets were both used for GSEA independently.

### Time-series analysis of differential gene expression

The DESeq2 package in R was utilized to identify genes that displayed differential expression patterns throughout the culture period. These genes were then designated as significant time sequence genes. For selection purposes, we chosen time sequence gene that had a false discovery rate (FDR) below 5% in DESeq2. To further analyze these genes, we employed Mfuzz package in R, which performs soft clustering based on the fuzzy c-means algorithm. The average TPM value of a single gene, considering replicates at each time point, was used as input for the Mfuzz clustering analysis. The number of clusters was set to 3 and the fuzzifier coefficient (M) was set to 1.5.

### Statistical analysis of data

We performed statistical analysis using GraphPad Prism version 9.0.0 software. One-way analysis of variance (ANOVA) or Student's t test was used to identify differences between groups. *p* < 0.05 was considered statistically significant. Experimental images were analyzed using ImageJ software. Unless otherwise stated, all quantitative data were expressed as the mean ± standard error of the mean (SEM). The experiment was conducted with a minimum of three independent replicates.

## Results

### Comparison of biological properties of hUC-MSCs cultured by microcarrier–bioreactors and planar flasks

**Fig. 1:**
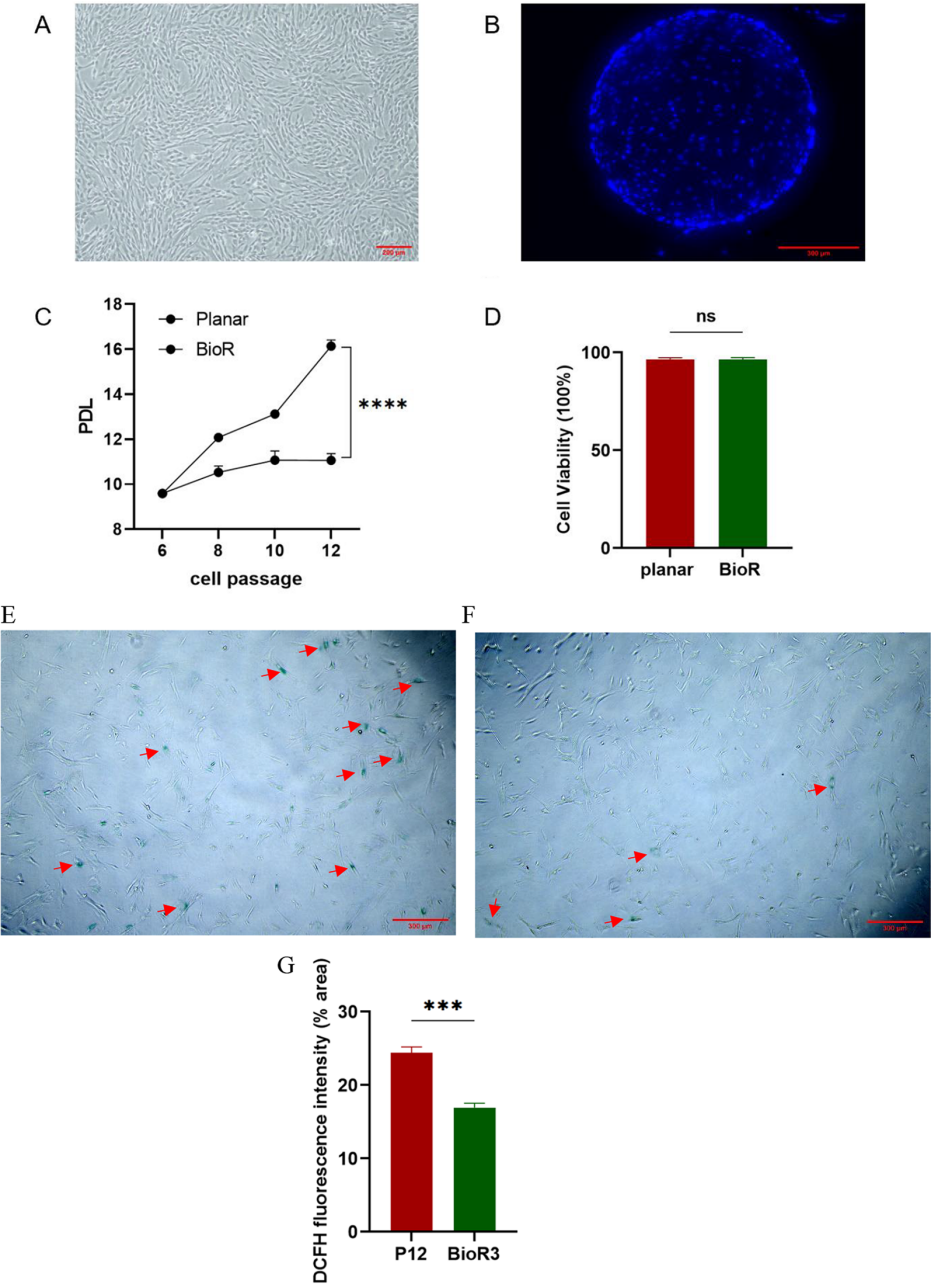
Growth of hUC-MSCs under two culture conditions. **A** Representative images of hUC-MSCs in planar; scale bar, 200 μm. **B** Representative images of hUC-MSCs in microcarrier; scale bar, 300 μm. Blue is the nucleus staining performed by DAPI. **C** PDL of cells in two culture methods. **D** Cell viability of the two culture methods. **E** β-Gal staining of P12 cells from planar culture; scale bar, 200 μm. **F** β-Gal staining of BioR3 cells from microcarrier–bioreactor culture; scale bar, 200 μm. **G** ROS levels of cells in two culture modalities. Data are presented as mean ± SEM. **p < 0.05

Cells grew in planar culture flasks and globular microcarrier as a single layer (Fig. 1A, B). An observation of the images reveals that hUC-MSCs grown under these two culture conditions exhibit a comparable morphology characterized by swirling, fibroblast-like adherent growth.

To compare the growth potential of hUC-MSCs amplified in the two conditions, the 6^th^ passage of cells (P6) continuously cultured in flasks from primary hUC-MSCs were used as seeds. Seed cells were divided into two parts, which were continuously cultured in planar and bioreactor separately. Starting with planar cultured P6, cells obtained by successive batches of transfer culture by the bioreactor were named as BioR1, BioR2, and BioR3. Since the planar culture was passaged once every 3 days and the bioreactor culture was transferred once every 6 days, the time required to reach P12 through continuous planar passage and BioR3 through continuous bioreactor transfer from P6 was equivalent. As shown in Fig. 1C, the PDL of P12 in planar culture was 11.2, while the PDL of BioR3 reached 16.139, which was 1.44 times higher than the former. So, our microcarrier–bioreactor culture process effectively improved cell proliferation. Cell viability in both cultures remained stable at > 95% (Fig. 1D).

Furthermore, microcarrier-amplified hUC-MSCs showed lower β-galactosidase activity (Fig. 1E, F) and lower ROS levels (Fig. 1G) compared to planar cultures, suggesting a younger status of bioreactor-cultured stem cells. In addition, hUC-MSCs amplified by microcarrier–bioreactors maintained good adhesion (Additional file 1: Figure S1).

### Effect of microcarrier–bioreactor culture on cell differentiation capacity

**Fig. 2:**
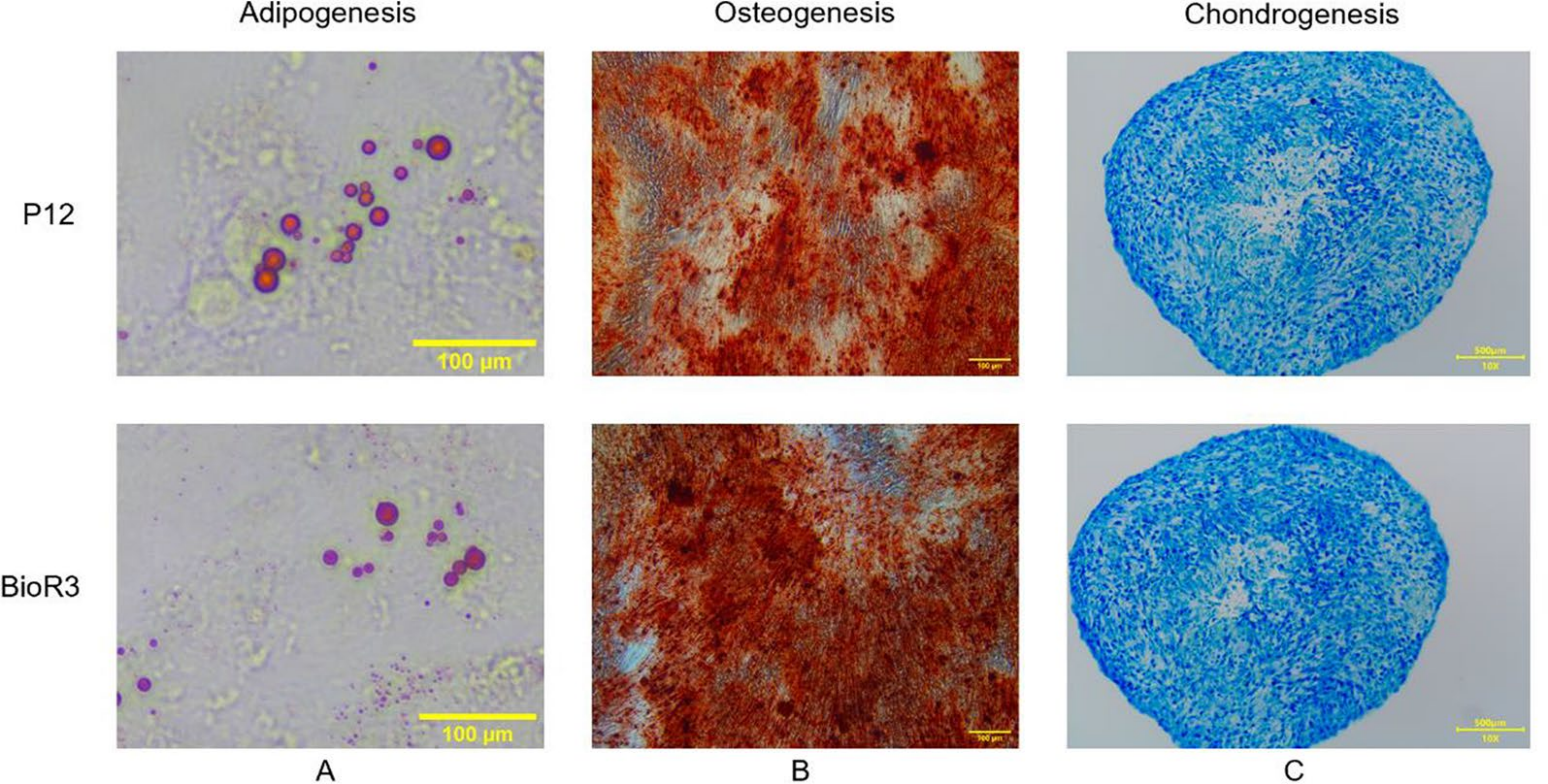
Differentiation potential of cells in both cultures. **A** adipogenesis; scale bar, 100 μm. **B** osteogenesis; scale bar, 100 μm. **C** chondrogenesis; scale bar, 500 μm

The effect of continuous culture on cell differentiation ability under the two culture methods was further studied (Fig. 2A–C). hUC-MSCs acquired from P12 and BioR3 were subjected to three-lineage induction differentiation. Notable, cells from both culture conditions demonstrated the retained ability to differentiate into lipid (Fig. 2A), osteogenic (Fig. 2B), and cartilage lineages (Fig. 2C). These results suggest that the utilization of microcarrier–bioreactor for cell culture does not alter the differentiation potential of the cells.

### Surface markers of hUC-MSCs under two culture conditions

**Fig. 3:**
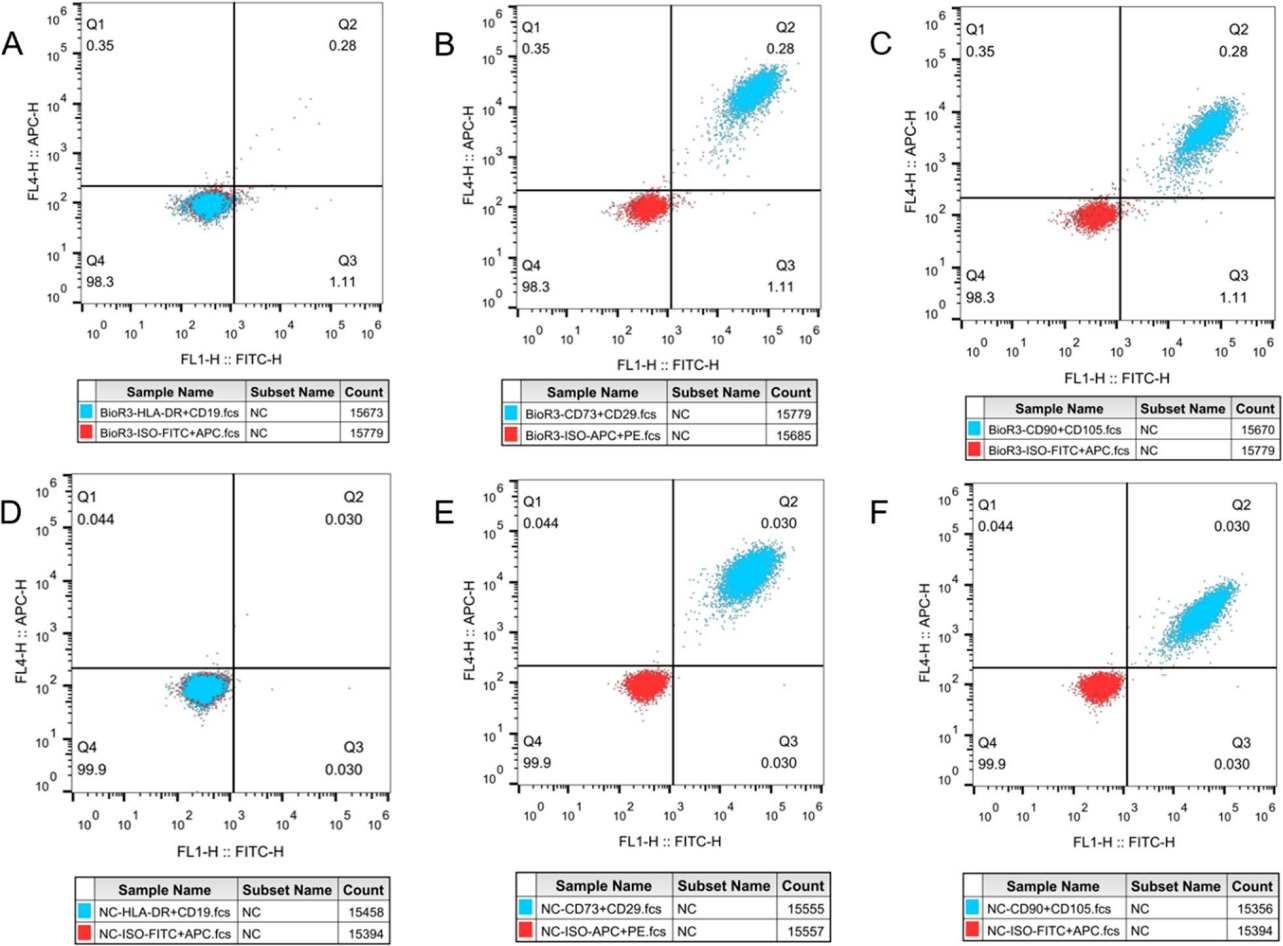
Changes in surface marker expression of hUC-MSCs in planar culture versus bioreactor culture. Surface labeling of hUC-MSCs was characterized using FACS analysis. **A** Negative cell markers of BioR3 (CD19, HLA-DR). **B** Positive cell markers of BioR3 (CD29, CD73). **C** Positive cellular markers of BioR3 (CD90 and CD105). **D** Negative cell markers for P12 (CD19, HLA-DR). **E** Positive cell markers for P12 (CD29, CD73). **F** Positive cellular markers of P12 (CD90 and CD105). Red indicates negative versus ISO, and blue indicates the corresponding surface marker

To compare the phenotypic characteristics of hUC-MSCs cultured in a bioreactor system and a planar system, the expression of representative stem cell surface markers was analyzed by using fluorescence-activated cell sorting (FACS). The levels of negative cell markers, CD19 and HLA-DR (Fig. 3A, D), and positive cell markers, CD29 and CD73 (Fig. 3B, E), CD90 and CD105 (Fig. 3C, F), were evaluated. The results showed that the positive surface markers of hUC-MSCs were all expressed above 98% in the bioreactor-cultured hUC-MSCs, while the negative surface markers of hUC-MSCs features were all expressed below 1%. Thus, there was no significant change in surface protein marker expression between planar culture and bioreactor-cultured hUC-MSCs (Fig. 3).

### Transcriptome data to assess the aging status of cells under two culture modalities

**Fig. 4:**
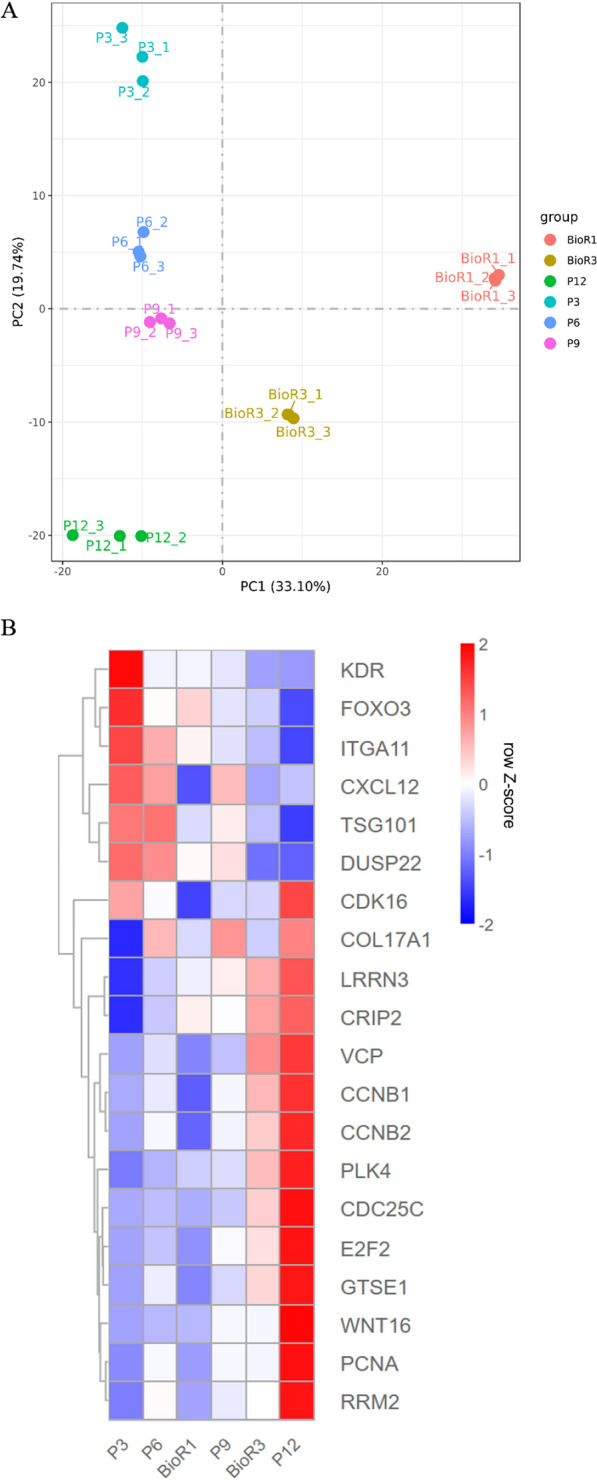
The RNA genome-wide sequence data of each group of hUC-MSCs were analyzed. **A** Global expression analysis using PCA. **B** Heat map of hierarchical clustering results showing aging-associated genes (rows) among hUC-MSCs in each experimental group. In contrast to P3, red and blue bars indicate genes that are up- and down-regulated, respectively. For all comparisons, changes in gene expression are described as heat maps

The principal component analysis (PCA) of transcriptome data from six samples sets revealed that the first two principal components (PC1: 33.1%, PC2: 19.74%) accounted for a total of 52.84% of gene expression variability (Fig. 4A). The PCA effectively separated the transcriptional characteristics between groups, confirming the reliability of the RNA-seq data quality for subsequent functional analysis.

The PCA diagram clearly demonstrates that the samples for the planar culture group (P3 to P12) align in a top-to-bottom order along the PC2 dimension, indicating a correlation between cell aging and the PC2 dimension. Additionally, the positioning of BioR1 between P6 and P9, as well as BioR3 between P9 and P12 along the PC2 axis, reflects the relative degree of aging in the two bioreactor cell samples (Fig. 4A). These findings are consistent with the results obtained from β-gal staining and ROS level determination, which indicate a lower degree of cellular senescence of BioR3 compared to P12 (Fig. 1E, F).

The transcriptome data were further combined with literature analysis to identify the representative genes representing aging. Twenty genes were selected, namely CCNB1, CCNB2, CDK1, KDR, FOXO3 [21], ITGA11 [22], CXCL12 [23], TSG101, DUSP22 [24], COL17A1 [25], LRRN3 [26], CRIP2 [26], VCP [27], E2F2 [28], GTSE1 [29], PLK4 [30], CDC25C [29], WNT16 [31], PCNA [32], RRM2 [33]. Heat maps were generated by plotting the expression levels of these senescence-associated gene population in six groups of samples (Fig. 4B). The genes were divided into two groups by the heat map, with increased gene expression levels observed in one group (the first 7 genes) with increasing generations of plane culture, while the other group (the last 13 genes) showed the opposite trend (Fig. 4B). Therefore, to some extent, the expression level of these genes reflects the senescence state of the cells. It was observed that the levels of senescence-related genes in BioR3 cells were between the planar culture P9 and P12 generations (Fig. 4B). This result also suggests that the cells harvested from the microcarrier–bioreactor culture are comparatively more youthful.

### Temporal sequence analysis and differential expression gene function enrichment analysis of cell transcriptome under two culture conditions

Our study collected six sets of samples (P3, P6, P9, P12, BioR1, BioR3), with three biological replicates in each group, to investigate changes in gene expression profiles between different cell generations and culture methods.

**Fig. 5:**
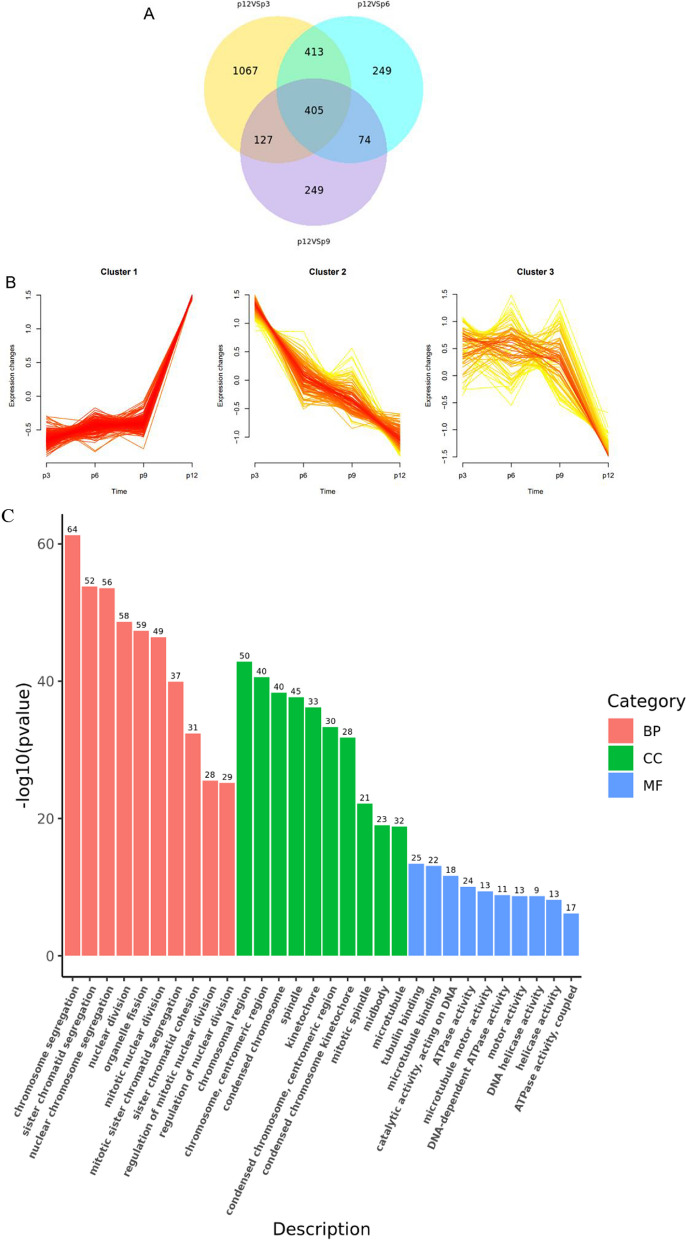

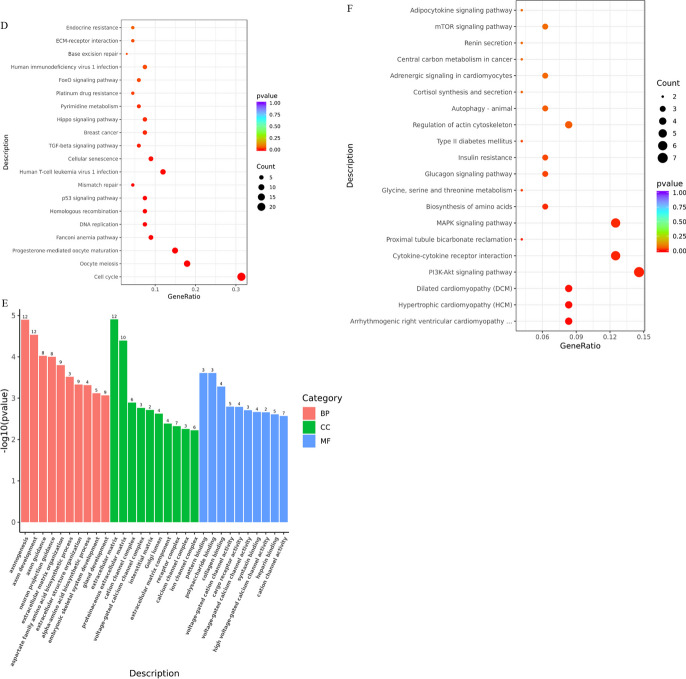
Time-series analysis of differentially expressed genes in plane culture. **A** Venn diagram shows the genes common to each generation compared to P12. **B** Cluster analysis of time sequence genes during planar culture. The x-axis represents each sample. **C**, **D** Cluster 1 upregulates the GO enrichment (**C**) and KEGG pathway enrichment (**D**) of the gene cluster. E–F: Cluster 2 downregulates GO enrichment (**E**) and KEGG pathway enrichment (**F**) of gene clusters. *p* < 0.05 and |log2FC|> 1.5

The phenotypes associated with cell aging (flat, enlarged form, etc.) were observed to increase during planar culture (Additional file 1: Figure S1). At the same time, transcriptome data revealed that the expression levels of certain genes gradually changed with increasing generations compared to those of P3 cells (Additional file 1: Figure S2). Therefore, it is plausible that the accumulation of differentially expressed genes (DEGs) during passages may play functional roles related to cell senescence. To explore this further, the transcriptomic data of P12 samples were compared with those of P3, P6, and P9, respectively. This analysis led to the identification of 405 common significantly DEGs (Fig. 5A). Subsequently, these 405 DEGs were subjected to expression time-series analysis, which resulted in the formation of three distinct cluster groups (Fig. 5B). Among these clusters, clusters 1 and 2, characterized by continuous upregulation and downregulation of gene expression, respectively, were considered closely associated with cellular senescence during planar culture and underwent further analysis.

The functional annotation analysis of Cluster1 revealed that many of the upregulated DEGs were enriched in biological processes (BP) related to chromosome, sister chromatid, nuclear chromosome, and nuclear division separation (Fig. 5C). In addition, further KEGG analysis indicated that the upregulated DEGs were significantly enriched in signaling pathways such as the cell cycle, DNA replication, cell aging, and p53 signaling pathways (Fig. 5D). Notably, the genes CCNB1, CDK1, and CCNB2 were jointly involved in the significant upregulation of these signaling pathways. Cyclin-dependent kinases 1 (CDK1), one of the important members of the protein kinase family, play a crucial role in regulating the cell cycle, activating checkpoints, and repairing DNA damage [32]. The key regulatory factors of the G2/M checkpoint in the cell cycle, cell cycle protein B1 (CCNB1), and cell cycle protein B2 (CCNB2), are important for the expression of pluripotent genes [34]. Additionally, CCNB1, CCNB2, and CDK1 are central genes in the p53 signaling pathway [35]. Research has shown that CDK negatively regulates DNA biosynthesis [32], while upregulation of the p53 signaling pathway leads to cell cycle arrest, ultimately resulting in senescence-associated heterochromatin foci and cellular senescence [35]. These findings suggest that the upregulated DEGs precisely regulate cell cycle processes.

The analysis of the GO term and KEGG pathway function annotations for Cluster 2 revealed that the downregulated DEGs were mostly enriched in phylogenetically related BP such as axonogenesis, axon development, axonal guidance, and neuronal projection guidance (Fig. 5E). Furthermore, the KEGG analysis demonstrated that disease-related pathways, PI3K-Akt signaling pathway, MAPK signaling pathway, cytokine–cytokine receptor interaction, and other signaling pathways were significantly enriched (Fig. 5F). The significant downregulated genes involved in these pathways were FGFR2 and KDR. The protein encoded by the FGFR2 gene is a member of the fibroblast growth factor receptor (FGFR) family and plays an important role in embryonic development and tissue repair [36]. FGFR, as an FGF receptor, is primarily responsible for transduction of FGF signals into RAS-ERK and PI3K-AKT signaling pathways and amplification [36]. KDR gene is an important vascular endothelial growth factor receptor in human body and also plays an important role in embryonic development and growth [37]. It has been observed that MSCs activate the FAK and Akt-mTOR-S6K1 signaling pathways by binding TGF-β1 to its specific receptors, which accelerates G/S cell cycle switching in order to stimulate cell proliferation [38]. In this regard, the downregulated DEGs for PI3K-Akt signaling pathway, calcium signaling pathway, and MAPK signaling pathway affect cell cycle conversion, resulting in decreased cell proliferation and ultimately accelerating cellular aging.

In order to reveal the genome expression profile responses to continuous microcarrier–bioreactor culture condition, the transcriptomic data of BioR3 were compared with that of P3, P6, and BioR1, respectively. The common DEGs obtained were used for temporal sequence analysis and resulted in three distinct cluster groups (Additional file 1: Figure S2A-B). GO enrichment and KEGG pathway analysis were performed on the continuous upregulated and downregulated gene clusters (Additional file 1: Figure S2C-F). The downregulated genes were mainly enriched in GO terminology related to phylogeny, consistent with the results from the data analysis of planar culture (Fig. 5E, F, Additional file 1: Figure S3C). Further analysis of KEGG signaling pathway showed that it was mainly clustered in MAPK signaling pathway, TGF-β signaling pathway, cytokine–cytokine receptor interaction, and other signaling pathways (Additional file 1: Figure s3D). Regarding continuously upregulated gene clusters, differences between the two culture methods were observed. The upregulated DEGs in microcarrier–bioreactor culture were mainly enriched in tight junctions signaling pathways associated with the extracellular matrix (Additional file 1: Figure S3E-F), while planar cultures were mainly enriched in cell cycle-related pathways (Fig. 5D).

When considering the results as a whole, it can be inferred that genes and signaling pathways associated with phylogeny are downregulated during aging in both culture conditions. However, unlike planar cultures, the expression of extracellular matrix (ECM)-associated genes and signaling pathways is continuously upregulated in microcarrier–bioreactor cultures. This finding is consistent with previous studies indicating that agitation regulates shear stress in suspension cultures, resulting in differential modulation of cytoskeleton/ECM tissue, proliferation, and metabolism-related pathways [39, 40].

### Transcriptome comparison of final harvested cells in two cultures

**Fig. 6:**
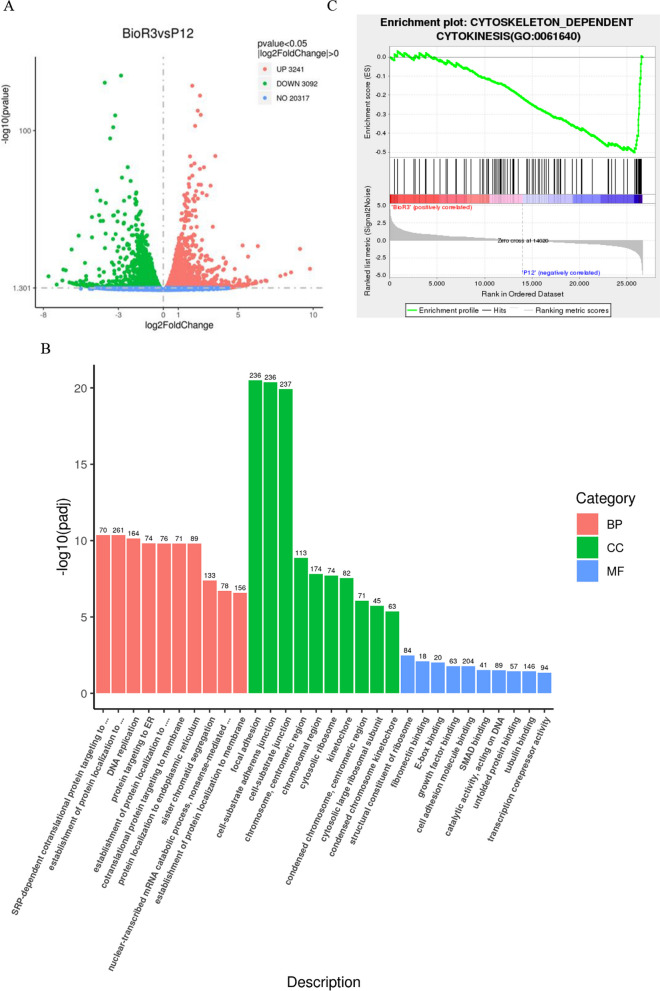
Transcriptome comparison between P12 cell generation cultured in plane and BioR3 cultured in bioreactor for three times. **A** DEGs volcano map. Upregulated genes are shown in red and downregulated genes are shown in green. *p* < 0.05, FC > 1. **B** GO enrichment analysis plot of BioR3vsP12. Each functional classification shows only the top ten terms and shows the number of DEGs associated with each term. **C** Gene set enrichment analysis (GSEA) showed significant negative enrichment of the "cytoskeleton-dependent cell division" gene set in P12 relative to BioR3 (normalized enrichment fraction [NES] =  − 2.495; *p* < 0.05)

To compare the major expression differences in cells obtained by culture for equal duration between the two methods, transcriptomic data of BioR3 and P12 were compared. BioR3 exhibited a total of 3241 downregulated genes and 3092 upregulated genes in comparison with P12 (Fig. 6A). GO enrichment analysis revealed that bioreactor culture affected pathways associated with many cellular components, including adhesion, cell–substrate adhesion, cell–substrate ligation, and more (Fig. 6B). To further confirm the effect of bioreactor culture on the cytoskeleton and cytoplasmic matrix, GSEA specifically focused on the expression of cytoskeleton-dependent cytokinesis gene set. The results revealed a significant decrease in the enrichment score of these genes in P12 compared with BioR3, indicating a distinct cytoskeletal organization between the two groups of cells (Fig. 6C). Both cytoskeleton and cadherin-related signaling pathways were downregulated in P12 group, possibly as a result of altered cell morphology, reduced cellular matrix adhesion, and recombinant cytoskeleton [41, 42]. Morphologically, BioR3 appeared to be more elongated and crescent-shaped than P12 (Additional file 1: Figure S1).

**Fig. 7:**
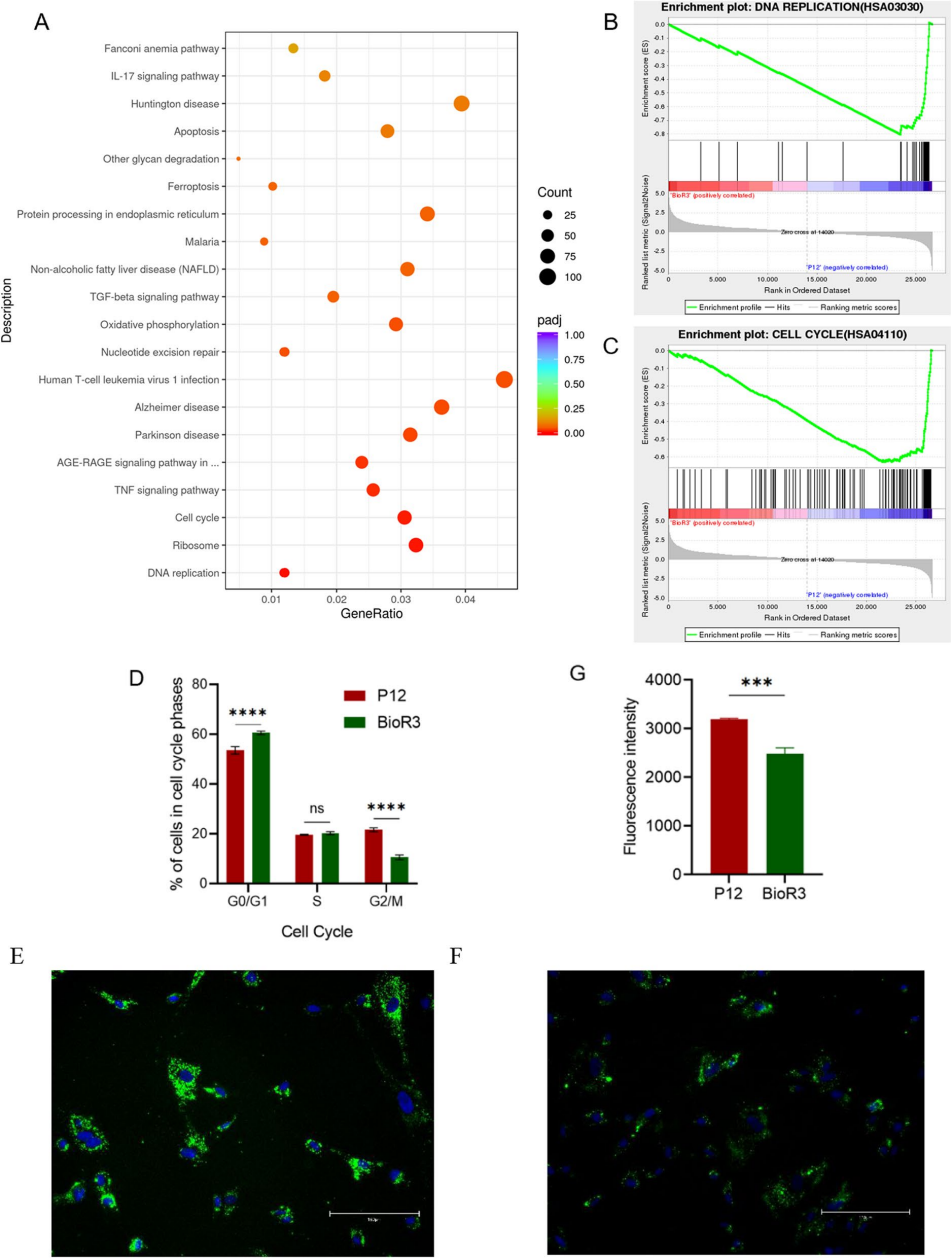
Cell cycle function analysis of P12 and BioR3. **A** KEGG enrichment analysis plot. **B** Gene set enrichment analysis (GSEA) showed significant negative enrichment of the "DNA replication" gene set in P12 relative to BioR3 (standardized enrichment fraction [NES] =  − 1.579; *p* < 0.05). **C** Cell cycle of BioR3 and/or P12 is analyzed by flow cytometry 48 h after seeding. Cells are gated according to forward scatter and side scatter to separate debris. Results are expressed as the percentage of cells gated at different cell cycle stages (*n* = 3). **D** Gene set enrichment analysis (GSEA) showed significant negative enrichment of the "cell cycle" gene set in P12 relative to BioR3 (standardized enrichment fraction [NES] =  − 1.637; *p* < 0.05). **E** PKH67 staining detects proliferation and division of P12 generation cells; scale bar, 150 μm. **F** PKH67 staining detects proliferation and division of BioR3 cells; scale bar, 150 μm. G: Average fluorescence intensity of cells under two culture conditions. Data are presented as mean ± SEM. ***p < 0.05, ****p < 0.01

Significant differences in gene expression involved in DNA replication and cell cycle pathways were observed in KEGG enrichment analysis of BioR3 and P12 cells (Fig. 7A). This finding was further validated through GSEA of DNA replication and cell cycle gene set. The analysis revealed enrichment in the gene regulatory processes associated with cell cycle frequency, rate, range, and direction in the P12 group. Additionally, the regulatory process of DNA replication was significantly downregulated (Figs. 7B, C). This suggests that during planar culture, genes involved in DNA damage response, cell cycle regulation, and apoptosis accumulated repetitive aging resulting from long-term subculture, in addition to genes associated with aging and senescence.

### Analysis of cell cycle function under two culture methods

Differences in cell cycle and division between the two cultures were suggested by transcriptome analysis. The cell cycle distribution of BioR3 and P12 was then analyzed (Fig. 7D). BioR3 cells exhibited a significant increase in the proportion of cells in the G0/G1 phase of the cell cycle compared to P12, while the G2/M phase decreased significantly. This indicates that bioreactor culture reduces the residence of cells in the G2/M phase and promotes cell division.

To further validate this result, we used PKH67-labeled cell membranes to detect in vitro proliferation of cells (Fig. 7E, F). The staining results showed that the cells cultured by the bioreactor carried a more dispersed fluorescence intensity (Fig. 7G). It also demonstrated that the cells harvested from the bioreactor culture had better proliferation ability than P12 generation cells.

## Discussion

The main objective of this study was to compare the performance and transcriptomic differences between hUC-MSCs from bioreactor and flask. It was found that hUC-MSCs obtained from both conventional planar and bioreactor cultures met the minimum MSCs criteria defined by ISCT, including plasticity adhesion, expression of selected surface markers, and trilineage differentiation capacity [43]. In this study, efficient amplification of hUC-MSCs was achieved through an established process based on self-made microcarriers for scale-up in a stirred bioreactor.

To investigate transcriptome changes induced by continuous culture and identify transcriptional differences between the two cultures, separate time-series analyses were conducted to identify genes associated with the aging process (Fig. 5). The expression dynamics of aging markers reported in the literature were also referred (Fig. 4B). It has been reported that decreased expression of cell adhesion molecules may lead to aging in stem cells, such as members of the integrin-family and related signaling pathways [44]. Integrin is a heterodimeric cell adhesion receptor consisting of "α" and "β" subunits capable of recognizing changes in the extracellular environment and regulating intracellular signaling and membrane-binding organelles [44]. Studies have found that the decreased expression of integrin ITGA3 during long-term passage of tonsil-derived mesenchymal stem cells (TMSCs) will reduce the phosphorylation of AKT on serine, thus leading to the aging of TMSCs [45]. However, this study found that ECM–receptor interactions and genes related to the PI3K-AKT signaling pathway (KDR, FOXO3, ITGA11) were continuously downregulated during long-term passage of hUC-MSCs and amplification of microcarrier–bioreactors (Fig. 4B), but ITGA3 showed nonlinear expression changes. Senescence genes may differ between MSCs sources and culture conditions, so related genes are not significantly represented in this study. The expression of some commonly used aging marker genes is significantly increased in late emergence passage (e. g. CDK1, WNT16) (Fig. 4B). Other biomarkers like TP53 and ATM, display nonlinear expression changes during continuous culture, making them unreliable predictors of aging state for hUC-MSCs. Therefore, in this study, aging manifests itself as the accumulative alterations in transcription levels of a group of aging-related genes.

Danielle M et al. proposed that hUC-MSCs exhibit minimal transcriptome drift when passaged early (P2) to mid-passage (P6-P8). However, transcriptome drift gradually increases by late passage (P9-P12) [20]. They also proposed that once the optimal hUC-MSCs amplification protocol has been developed in bioreactor cultures, the transcriptome drift states described for static cultures will need to be re-evaluated [20].

In order to gain further insights into the process changes occurring during continuous cell culture in a microcarrier–bioreactor, the comparison between transcriptome of BioR1 and P9 (BioR vs. P9) was also analyzed. Similar to the observations made in BioR3 vs. P12, the GO enrichment analysis revealed that bioreactor culture had comparable effects on pathways related to various cellular components, such as adhesion, cell–substrate adhesion, and cell–substrate connectivity (Additional file 1: figure s4). Additionally, the GSEA of the focal adhesion gene set demonstrated a significant reduction in the enrichment score of genes in P9 compared to BioR1, indicating distinct cytoskeletal organization between the two cell groups (Additional file 1: figure S4). Notably, in the KEGG enrichment analysis of BioR1 and P9 cells, significant differences in gene expression were primarily observed in the adherence junction pathway (Additional file 1: figure S4). This suggests that changes in cell gene expression profiles during the transition from planar culture to microcarrier–bioreactors occur gradually and accumulate over time.

A wide range of yields have been reported in the literature on hMSCs microcarrier culture. Elseberg et al. conducted a study on hMSCs expansion in a stirred tank bioreactor system. They cultured the cells in a bioreactor containing 1.7 L of medium for 6 days without adding any feed. At the end of the culture period, a 7.2-fold expansion was achieved [46]. In this study with a similar duration and no feeding, an eightfold expansion was achieved in a 200 ml culture volume, indicating a significantly improved amplification efficiency. It should be noted that the expansion efficiency of cells can be further improved by modifying the culture process, such as changing the feeding method, culture volume, or culture time [47–49]. For example, Cierpka et al. performed batch culture of human mesenchymal stem cells in a stirred tank bioreactor and achieved 6.9-fold expansion at the end of the culture (6 d) [50]. In order to further improve cell expansion, Cierpka et al. extended the culture time and used slow agitation (35 rpm) in the early stage of culture to promote good cell adhesion. During 12 days of cultivation, the stirrer speed was increased to 75 rpm. The increase in stirrer speed was combined with a medium feed (1 L DMEM-LG after 7 d, 0.4 L DMEM-HG after 11 d). This fed-batch strategy gave a 6-time higher expansion factor when compared to batch cultivations [50]. These studies demonstrated significant variations in cell expansion levels, which arose from various factors including initial cell properties, microcarrier characteristics, medium components, and differences in process parameters and culture conditions [51]. As a result, there is still huge ample room for optimizing the temporal and spatial efficiency of hMSCs expansion through bioreactor culture methods.

Our findings suggest that while cells expanded by the microcarrier–bioreactor had a higher expansion efficiency, they also maintained a more youthful state and possible better cell quality compared to cells from the same origin and cultured in flasks for equivalent generations. Through transcriptome analysis, the mechanisms are speculated to be involved in the cytoskeleton, cell cycle, and DNA replication. Bioreactor culture has been found to promote cell–cell interactions (e.g., Notch pathways), ECM secretion, and enrichment in various cell types [52–54]. Furthermore, consistent with our study, Lam et al. discovered, using an integrated systems-level multiomics approach, that the pathway of significant enrichment in microcarrier–bioreactor cultures was mainly associated with cytoskeletal and ECM interactions, as well as signaling pathways involved in the regulation of growth, development, and metabolism [5]. During the transition from static plane to dynamic expansion, the microcarrier-based expansion process introduces shear stress through agitation and alters the topographic and surface environment to which the cells are exposed [39]. Previous studies have shown that cell behaviors such as cell motility, cell adhesion, and shape are influenced by surface chemistry, topography, and mechanical stimuli [55, 56]. Additionally, the secretion of extracellular vesicles (EVs) has been found to increase in bioreactor culture, suggesting that the impact of bioreactor culture on hMSC-EVs biogenesis may come more from enhanced cell–cell interactions and ECM enrichment, as well as associated intracellular signaling pathways [42].

In terms of differentiation of hUC-MSCs, we tested the differentiation ability of cells cultured in microcarrier–bioreactors and found that they had differentiation capabilities comparable to planar cultures for adipogenesis, osteogenesis, and chondrogenesis (Fig. 2). Similarly, Lam et al. found that the chondrogenic differentiation ability of cells cultured in a stirred suspension bioreactor with microcarriers was enhanced [5]. Studies have shown that the enhancement of stem cell cartilage formation potential may be attributed to the unique features of microcarrier-stirred suspension bioreactors, including microcarrier surface features and their stirring properties [57, 58]. To compare the cartilage-forming differentiation capacity of cells further quantitatively from the two cultures, the expressions of cartilage-related marker genes, including SOX9, ALPL, COL2A1, RUNX2, were extracted from transcriptome data (Additional file 1: Figure S5) and analyzed. The results showed that the expression level of related chondrogenic genes was enhanced in microcarrier–bioreactor culture, which was consistent with previous studies.

Studies have shown that MSCs from elderly donors have significantly reduced or no effect on treatment [59]. Therefore, overcoming the barrier of reduced therapeutic efficacy caused by aging is a huge hurdle. Transcriptome data analysis uncovered notable alterations in signaling pathways linked to cellular senescence during long-term passage replication. Specifically, the cellular senescence and p53 signaling pathways, along with other signaling pathways, exhibited significant upregulation. Conversely, the PI3K-Akt signaling pathway, MAPK signaling pathway, and various other signaling pathways were significantly downregulated (Fig. 5C–F). Therefore, it is of interest to explore whether modulating signaling pathways can enhance cell proliferation efficiency and delay cellular aging. More and more researchers investigated the potential of incorporating specific compounds to improve cell proliferation efficiency and delay the aging process [60]. An example of such a compound is resveratrol, a natural compound known for its ability to activate sirtuin-1 (SIRT1), an NAD-dependent deacetylase enzyme. Resveratrol has been reported to diminish the cellular aging process [61]. SIRT1 has been shown to positively regulate cell survival, as well as responses to stress and inflammation through non-histone targets such as p53, FOXOs, and NF-kB [62]. In addition, coenzyme Q10 (CoQ10) was reported as a strong antioxidant that can inhibit lipid peroxidation and scavenge free radicals, effectively reducing oxygen radical levels due to long-term passage replication, thereby alleviating cellular aging [63–65]. Studies have found that CoQ10 has anti-apoptotic and antioxidant effects on H_2_O_2_-induced death of bone marrow mesenchymal stem cells by activating the Nrf-2/NQO-1 pathway in vitro [66]. Therefore, relevant compounds for exogenous addition can be identified to alleviate the condition of cell aging and improve the quality of cells expanded in vitro through follow-up research.

## Conclusion

In this study, a microcarrier–bioreactor-based expansion method for hUC-MSCs was proved to meet the efficiency and quality requirements for stem cell therapy. The findings of this study contribute to our understanding of aging and cellular responses to different culture methods. The acquired knowledge may be utilized to develop strategies aimed at delaying cellular aging and enhancing stem cell function through the regulation of gene expression. Consequently, the implementation of a high-quality, efficient, and controllable in vitro expansion process for stem cells is expected to greatly advance the clinical application of stem cell therapy.

### Supplementary Information

**Additional file 1.** **Figures S1–S5.**

## Data Availability

The data used to support the findings of this study are available from the corresponding authors upon request. The RNA-seq data have been deposited in NCBI’s Gene Expression Omnibus and are accessible through GEO Series GSE233634 (https://www.ncbi.nlm.nih.gov/geo/query/acc.cgi?acc=GSE233634).

## Abbreviations

hUC-MSCs: Human umbilical cord mesenchymal stem cells
MSCs: Mesenchymal stem cells
GVHD: Graft-versus-host disease
hMSCs: Human mesenchymal stem cells
RNA-seq: RNA sequencing
PDL: Cell population-doubling level
PFA: Paraformaldehyde
SA-β-gal: Senescence-associated β galactosidase
ROS: Reactive oxygen species
GSEA: Gene set enrichment analysis
ANOVA: Analysis of variance
FITC: Fluorescein isothiocyanate
APC: Allophycocyanin
PCA: Principal component analysis
BP: Biological processes
CDK1: Cyclin-dependent kinases 1
CCNB: Cell cycle protein B
AMPK: Adenosine 5’-monophosphate (AMP)-activated protein kinase
FGFR: Fibroblast growth factor receptor
FAK: Focal adhesion kinase
mTOR: Mammalian target of rapamycin
ECM: Extracellular matrix
TMSCs: Tonsil-derived mesenchymal stem cells (TMSCs)
EVs: Exosomes
SIRT1: Sirtuin-1
CoQ10: Coenzyme Q10
Nrf-2: Nuclear factor E2-related factor 2
NQO-1: Quinone oxidoreductase
