# Supplementary material for: Efficient expansion and delayed senescence of hUC-MSCs by microcarrier–bioreactor system
Source: Stem Cell Res Ther. 2023 Oct 4;14:284. doi: 10.1186/s13287-023-03514-1 (PMC10552362; doi:10.1186/s13287-023-03514-1)
Supplement: Supplementary file 1 — Additional file 1. Figures S1–S5. [file 13287_2023_3514_MOESM1_ESM.pdf]

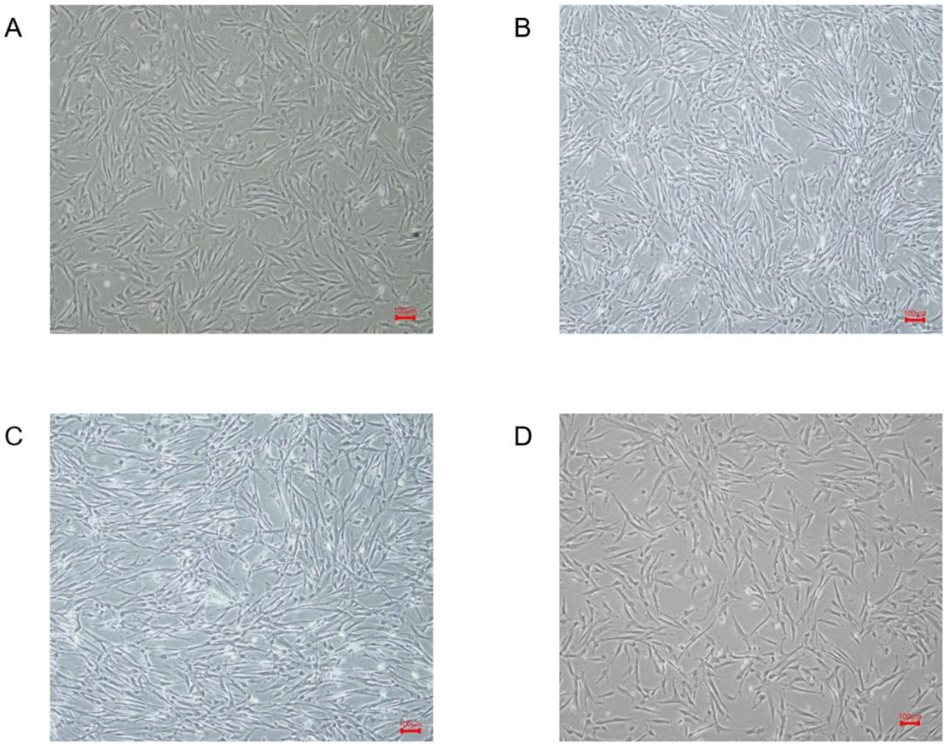

Supplementary Figure 1: Growth status of cells under two culture methods. A-C: Cell state of planar culture P6, P9, P12. D: Cell state cultured by microcarrier reactor. Related to Figure 1.

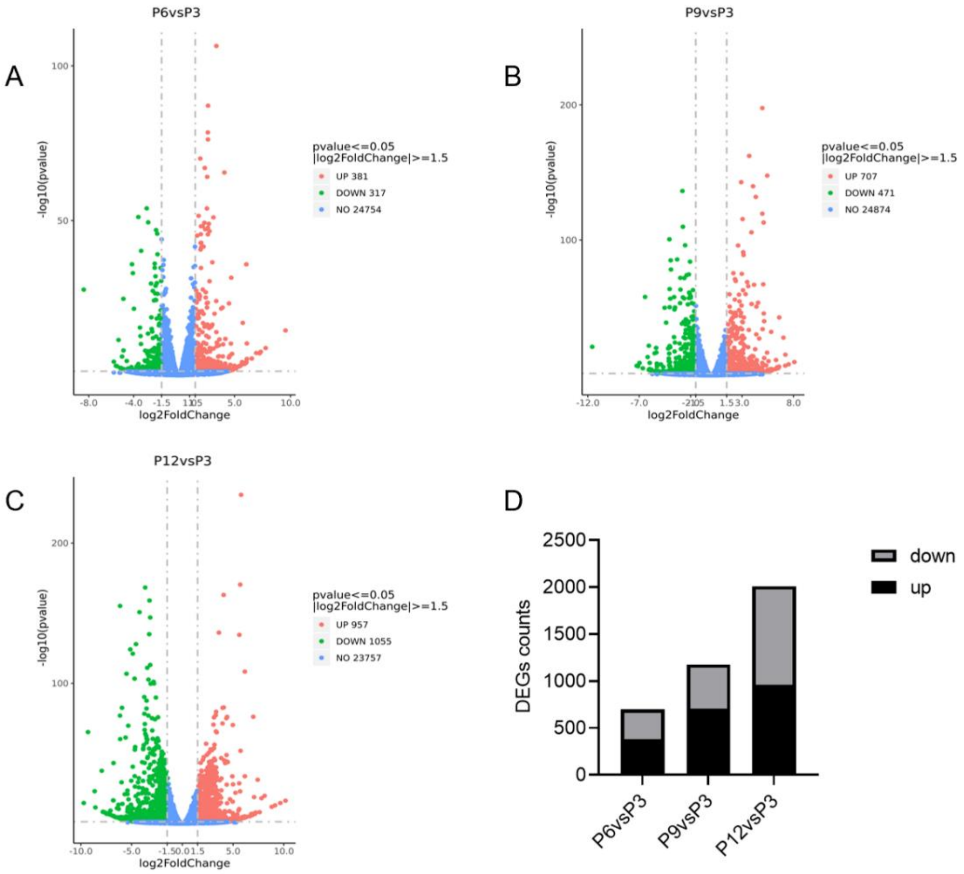

Supplementary Figure 2: Change in number of differentially expressed genes (DEGs) in P3 hUC-MSCs by generation in planar culture. A: Volcano map in P6vsP3. B: Volcano map of P9vsP3. C: Volcano map of P12vs P3. D: The histogram shows the change in the number of DEGs in each generation and P3 hUC-MSCs. Up-regulated genes are shown in red and down-regulated genes are shown in green. $p<0.05$ and $|log2FC|>1.5$. Related to Figure 5.

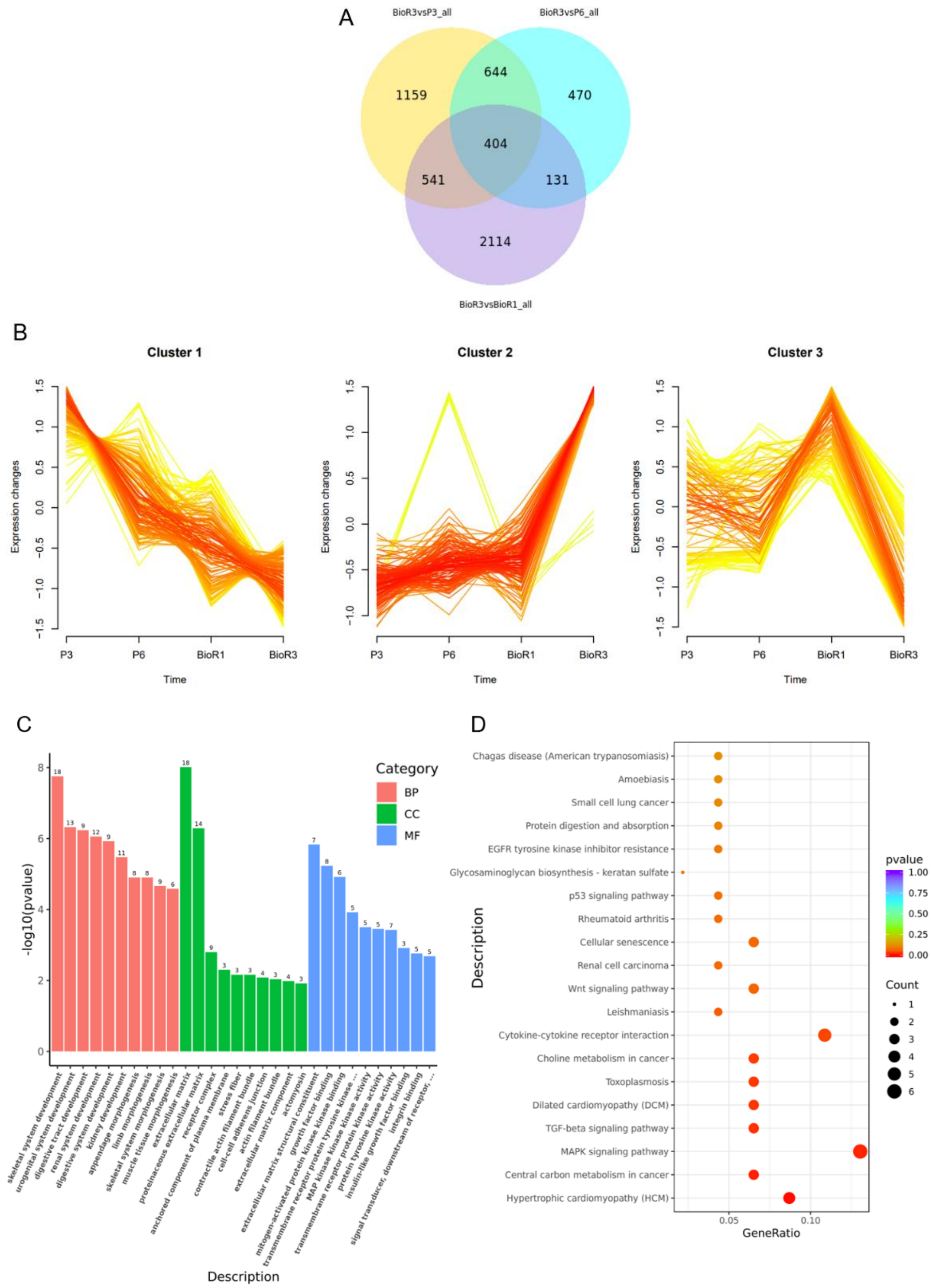

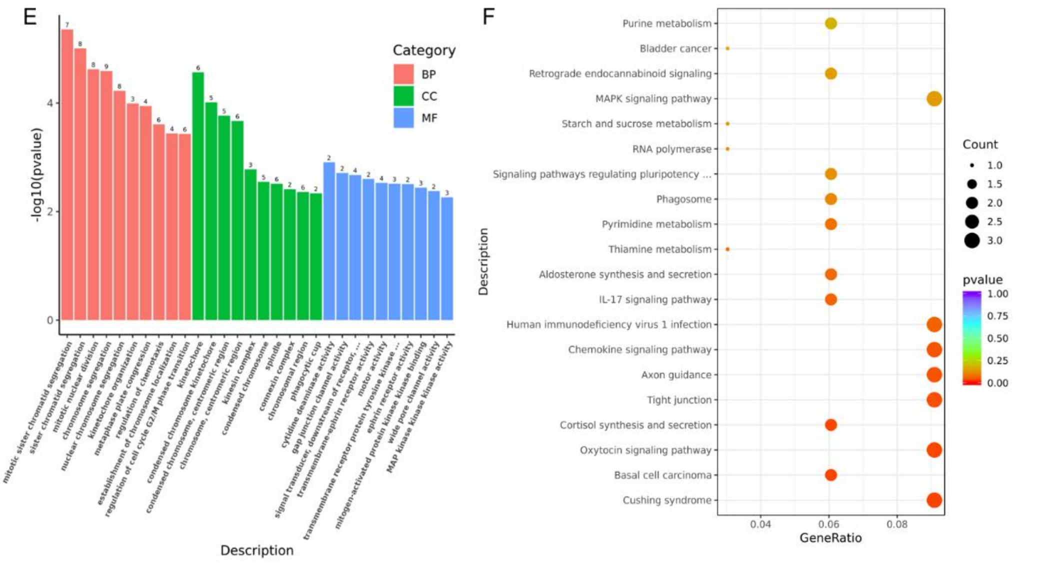

Supplementary Figure 3: Time series analysis of differentially expressed genes in microcarrier reactor culture. A: Venn diagram shows genes common to each generation compared to BioR3. B: Cluster analysis of time sequence genes during microcarrier reactor culture. The x-axis represents each sample. C-D: Cluster1 down-regulates GO enrichment of the genome with KEGG pathway enrichment. E-F: Cluster2 upregulates GO enrichment of the genome with KEGG pathway enrichment. Related to Figure 5.

A

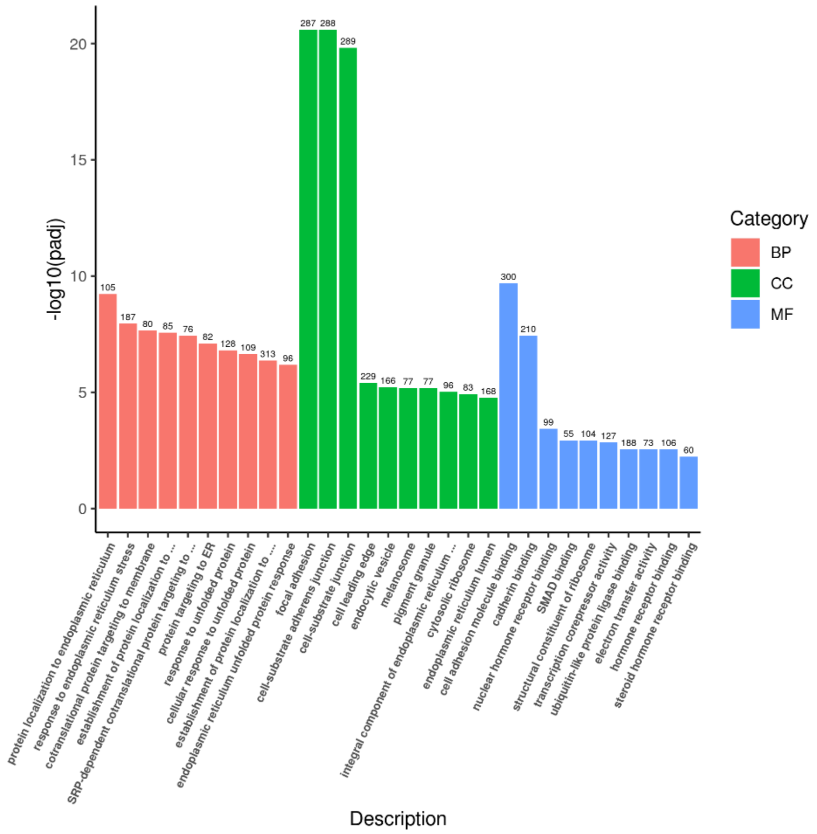

B

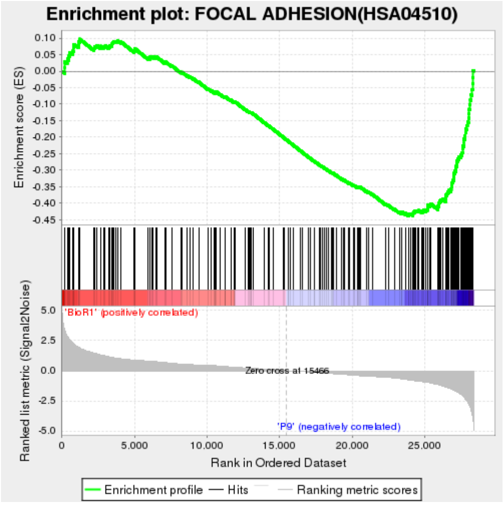

C

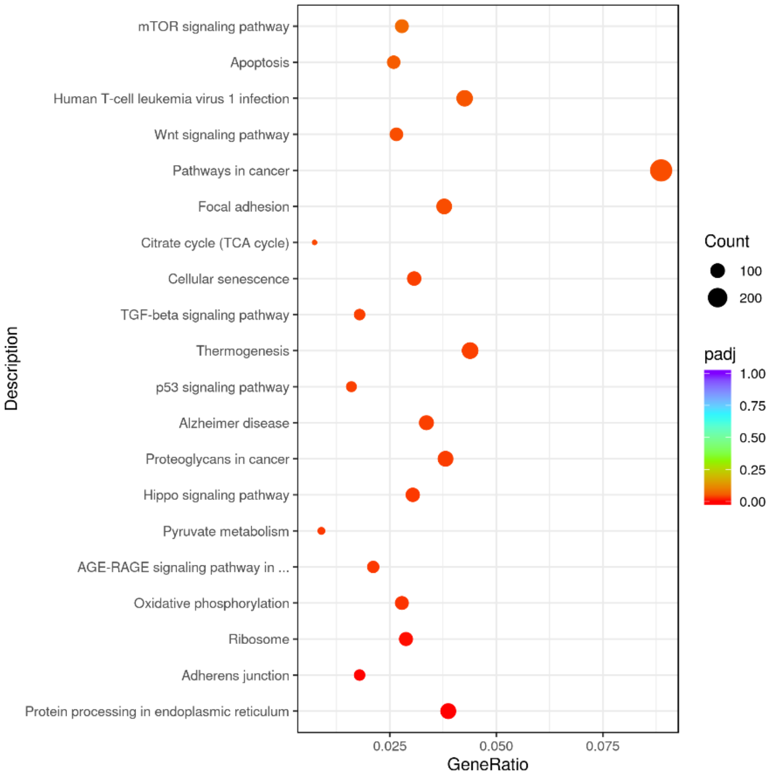

Supplementary Figure 4: Transcriptome comparison between P9 cell generation cultured in plane and BioR1 cultured in bioreactor for three times. A: GO enrichment analysis plot of BioR1vsP9. Each functional classification shows only the top ten terms and shows the number of DEGs associated with each term. B: Gene set enrichment analysis (GSEA) showed significant negative enrichment of the "focal adhesion" gene set in P9 relative to BioR1 (normalized enrichment fraction [NES] = −1.805; $p<0.05$). C: KEGG enrichment analysis plot of BioR1vsP9.

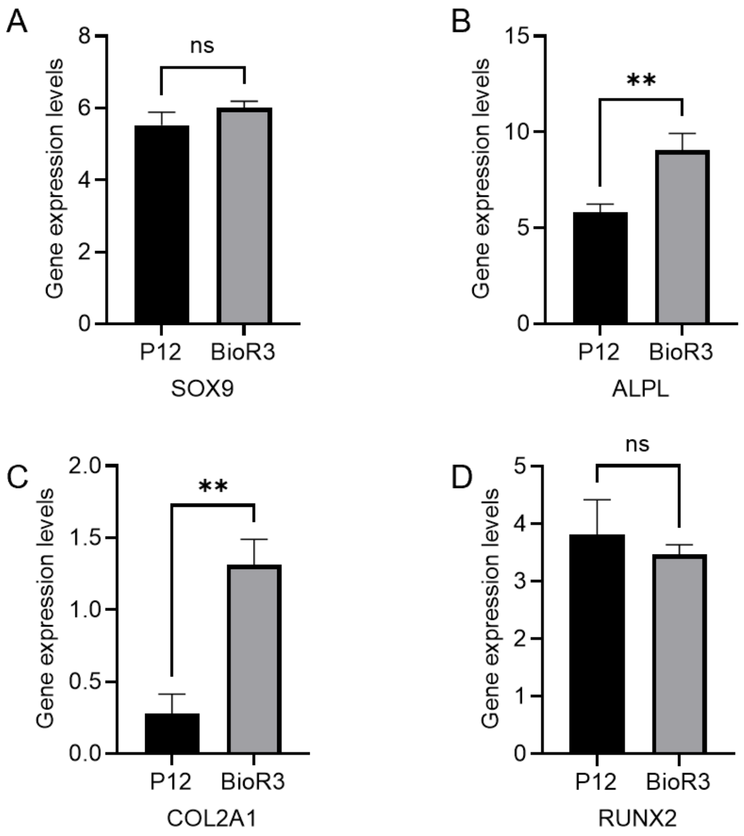

Supplementary Figure 5: Expression levels of cartilage-related marker genes. A: SOX9. B: ALPL. C: COL2A1.D: RUNX2. Data are presented as mean ± SEM. **p< 0.01.
